# A Machine Learning Approach for Modelling Cold-Rolling Curves for Various Stainless Steels

**DOI:** 10.3390/ma17010147

**Published:** 2023-12-27

**Authors:** Julia Contreras-Fortes, M. Inmaculada Rodríguez-García, David L. Sales, Rocío Sánchez-Miranda, Juan F. Almagro, Ignacio Turias

**Affiliations:** 1 Laboratory and Research Section, Technical Department Acerinox Europa S.A.U., 11379 Los Barrios, Spain; rociosanchezmiranda@gmail.com (R.S.-M.); juan.almagro@acerinox.com (J.F.A.); 2 Department of Materials Science Metallurgical Engineering and Inorganic Chemistry, Algeciras School of Engineering and Technology, Universidad de Cádiz, INNANOMAT, IMEYMAT, Ramón Puyol Ave., 11202 Algeciras, Spain; david.sales@uca.es; 3 MIS Group, Department of Computer Science Engineering, Algeciras School of Engineering and Technology, University of Cádiz, Ramón Puyol Ave., 11202 Algeciras, Spain; inma.rodriguezgarcia@uca.es (M.I.R.-G.); ignacio.turias@uca.es (I.T.)

**Keywords:** stainless steel, strain hardening, cold-rolling curves, machine learning, intelligent modelling, artificial neural networks

## Abstract

Stainless steel is a cold-work-hardened material. The degree and mechanism of hardening depend on the grade and family of the steel. This characteristic has a direct effect on the mechanical behaviour of stainless steel when it is cold-formed. Since cold rolling is one of the most widespread processes for manufacturing flat stainless steel products, the prediction of their strain-hardening mechanical properties is of great importance to materials engineering. This work uses artificial neural networks (ANNs) to forecast the mechanical properties of the stainless steel as a function of the chemical composition and the applied cold thickness reduction. Multiple linear regression (MLR) is also used as a benchmark model. To achieve this, both traditional and new-generation austenitic, ferritic, and duplex stainless steel sheets are cold-rolled at a laboratory scale with different thickness reductions after the industrial intermediate annealing stage. Subsequently, the mechanical properties of the cold-rolled sheets are determined by tensile tests, and the experimental cold-rolling curves are drawn based on those results. A database is created from these curves to generate a model applying machine learning techniques to predict the values of the tensile strength (*Rm*), yield strength (*Rp*), hardness (*H*), and elongation (*A*) based on the chemical composition and the applied cold thickness reduction. These models can be used as supporting tools for designing and developing new stainless steel grades and/or adjusting cold-forming processes.

## 1. Introduction

Stainless steels are hardened through diverse mechanisms depending on the steel family, with the strain hardening phenomenon being the most studied [1,2,3]. This implies changes in the mechanical properties when the material is cold-formed: tensile strength (*Rm*), yield strength (*Rp*), and hardness (*H*) are increased, while elongation or ductility (*A*) is reduced. Consequently, the mechanical behaviour, and, thus, the formability, during cold forming operations for a given stainless steel depends on this strain hardening phenomenon, which is influenced mainly by the chemical composition and forming process parameters, including the cold strain level, strain rate, and temperature, among others. Furthermore, the achieved mechanical properties after the final annealing treatment of a given stainless steel are a function of this strain hardening phenomenon. This means that the strain-hardened state before the final annealing treatment contributes to the final values of *Rm*, *Rp*, and *A* of the material.

To understand how these variables affect the mechanical behaviour and, thus, the mechanical properties of a cold strain-hardened stainless steel, large batches of tensile tests until fracture of a broad selection of cold-rolled materials must be fulfilled to build the known as cold-rolling curves. These graphs depict the evolution of the mechanical properties *Rm*, *Rp*, *A*, and *H* against the cold thickness reduction, which is utilised for the definition of the process window of a lot of cold forming processes, including cold rolling, for the design of new steels or the adjustment of conventional ones. Nonetheless, this is both an expensive and long-term procedure. To overcome these drawbacks, the development of computer technology over the last two decades has offered the opportunity to undertake this kind of study in an economical and faster way, thanks to machine learning methods [4,5]. A wide list of studies supports the use of these predictive techniques to estimate the mechanical behaviour of stainless steel under different states or conditions.

In the field of austenitic stainless steel, a number of works have been published during the last few years. Some examples of these are the approach conducted by Susmikanti and Sulistyo [6] to predict the strain hardening of the austenitic stainless steel under several cold strain levels using genetic algorithm and artificial neural networks (ANNs); the modelling of flow stress curves of austenitic stainless steel AISI 304 and 316 in dynamic strain ageing regimen made by Krishnamunthy et al. [7], Bahrami et al. [8] and Kumar et al. [9] applying the predictive methodology of ANNs; the design of models by Wand et al. [10] to predict the mechanical properties at room temperature including the *Rm* and *Rp* of the austenitic stainless steel AISI 304, 316, 321, and 347 as a function of the chemical composition, heat treatment, and test temperature; or the development of the model by Ono and Miyoshi [11] to predict the *Rm* and *A* from other mechanical properties like *E* or *Rp* among others for the austenitic AISI 304L and 316L. In addition to these, another important demonstrated use of the machine learning techniques is the prediction of the mechanical properties at elevated temperatures, as shown in the studies about the austenitic stainless steel AISI 304 performed by Kanumuri et al. [12] and the grades AISI 304L and 316L made by Desu et al. [13], both of which estimate the value of *Rm*, *Rp*, *A*, strain hardening exponent (*n*) and strength coefficient (*K*) as a function of the temperature, in the range from 50 °C to 650 °C, and the strain rate, for the three values of 0.0001, 0.001, and 0.01 s^−1^. Finally, another interesting application of the ANNs is the model designed by Forouzan et al. [14] to predict the appropriate annealing treatment conditions for the reversion of the martensite into austenite, which is an important tool for the design of a new AISI 304L austenitic with improved mechanical properties thanks to the grain refining microstructure obtained by this reversion mechanism. 

Concerning the family of ferritic stainless steel, multiple authors support the use of machine learning techniques as a powerful tool to predict the mechanical behaviour of these steel grades. One of them is the modelling performed by Honysz [15,16,17] to determine the values of *Rp*, *Rm*, *A*, and *H* of rolled and forged ferritic stainless steel with a content of Cr between 10 and 14% as a function of the chemical composition and the heat treatment conditions. Other authors that have demonstrated good prediction of the mechanical properties of the ferritic by machine learning methods are Ono and Miyoshi [11], through the development of a model to predict the *Rm* and *A* from other mechanical parameters, like *E* or *Rp*, amongst others, for several varieties of stainless steel, among them the ferritic AISI 430. Furthermore, other research implemented by Mamun et al. [18,19] employs the machine learning methodology to estimate the creep rupture strength as a function of the chemical composition, test temperature, and time for a ferritic stainless steel with 9–12% Cr.

In the case of the duplex stainless steels, which are currently being introduced in the market and under continuous development for the design of new grades with improved properties, the ANNs methodology is also being applied to evaluate the mechanical properties of this stainless steel family with good results. For instance, it is the case of the developed model by Thankachan et al. [20] to estimate the *Rm* under casting conditions for the standard duplex S32205 [21], the modelling of the hot plastic flow curves of the super duplex stainless steel S32507 [21] by Contini Jr. and Balancin [22], the prediction of the hardness of the ferrite during low-temperature ageing of duplex stainless steel by Karlsson and Giard [23], the design of a model to estimate the *Rm* and *A* from other mechanical properties such as *E*, *Rp*, etc. of the duplex S32205 by Ono and Miyoshi [11], or the modelling of the impact energy of as cast duplex stainless steel based on its chemical composition by Thankachan and Sooryaprakash [24]. 

Among the several machine learning techniques that have been used by the previously listed authors, ANNs are the most used. They consist of a series of process elements organised in layers (input layer, hidden layers, and output layer) connected by weights whose values are typically calculated using a backpropagation algorithm [25]. ANNs have universal approximation capabilities [26] and are used in a wide range of fields. Vanem et al. [27] used statistical together with machine learning methods to design a model dealing with environment-known parameters. Sajjan et al. [28] used machine learning (ML) to identify hidden but pertinent patterns within given chemistry data. Al Haj Ali [29] applied similar methodological techniques in the field of stainless steel, using the Bonferroni post hoc test for pairwise comparisons and the ANOVA test to disclose any statistical significance of differences between the groups. The authors in [30,31,32,33,34] utilised non-linear machine learning models such as ANNs to predict the pitting corrosion resistance of the austenitic stainless steel AISI 316L as a function of the environmental conditions: pH, temperature, and Cl concentration. Ruiz et al. [35] made use of machine learning to evaluate the inclusion content of clean steel manufacturing by electric arc furnace and rolling as a function of manufacturing and non-manufacturing variables. Further, Han et al. [36] applied a vector machine model (SVM) for pellet metallurgical properties forecasting to improve the evaluation efficiency of this kind of raw material used in additive manufacturing.

In this particular area of study, the majority of published research has focused exclusively on the prediction of the mechanical properties of traditional stainless steels, such as the austenitics AISI 304 and 316, the ferritic AISI 430, and the duplex S32205. The factors being examined are typically the chemical composition, strain temperature, or strain rate, with no consideration of the strain state. However, given that the strain state of a given steel has a direct impact on its mechanical behaviour during any cold-forming operation, it is worth analysing the effect of this variable on the mechanical properties. Therefore, the purpose of this paper is to investigate the influence of the cold thickness reduction and the chemical composition on the mechanical properties of a wide range of stainless steels, going beyond the standard grades previously mentioned. The study aims to model different mechanical properties as a function of the chemical composition and the percentage of cold-working reduction of the stainless steels, comparing a linear model with a non-linear machine learning model. The approach presented here is based on the use of machine learning techniques to get linear or nonlinear internal relationships from empirical data. The introduction of this computational model in the industry could be useful for the reduction of manufacturing costs of materials, not only for the design of new alloys but also for the material selection, the definition of cold forming process conditions, the improvement of the quality of stainless steel and the prevention of serious damage to the environment or public safety, all of this thanks to the capability of the model of the present work to predict the mechanical behaviour of multiple varieties of stainless steel under strain hardening condition, advantages which have been already expressed by multiple authors such as Thike et al. [37], Goel et al. [38], Huang et al. [39] or Mouellefe et al. [40]. 

Furthermore, the selected materials for this study are defined together with the chemical composition and the experimental procedure to obtain the mechanical properties of the cold-rolled materials that have been used to build the database. Moreover, the test methods that have been considered in the modelling are described in the next sections. Subsequently, the main results are collected by comparing the results obtained from several test methods regarding the mean square error (*MSE*) and the correlation coefficient (*R*). Finally, the discussion of these results and the main conclusions are collected.

## 2. Materials and Methods

### 2.1. Materials

A total of seventeen stainless steel grades, which correspond to austenitic, ferritic, and duplex families, were included in this experimental work to have a wide process window with respect to the analysed range of chemical composition. For each steel grade, to ensure the repeatability of the results, two industrial sheets were selected with a thickness between 3.0 and 5.0 mm and after the hot-rolling and intermediate annealing stages, implying 1D mill finishing, hot rolling, annealing, and removing the mill scale according to the European Standard EN-10.088-2 [21]. The materials under study and their average chemical composition are summarised in Table 1, where the letter “A” is used to identify the austenitic samples, “F” for the ferritic ones, and “D” in the case of duplex grades.

The selection of austenitic stainless steel includes the standard AISI 304/EN-1.4301 grade, which is characterised by medium values of Ni and Cr, 8% and 18%, respectively; the AISI 301/EN-1.4310 steel, which is alloyed with C to increase the mechanical properties; the AISI 316L/EN-1.4404 type with higher Ni and Mo content, 10% and 2%, respectively, to improve the corrosion resistance; and the super-austenitic grades AISI 310S/EN-1.4845, AISI 309S/EN-1.4833 and AISI 904L/EN-1.4539, which are alloyed with Cr, Ni, Mo, and/or Cu to increase the mechanical behaviour, the corrosion resistance, and the oxidation at high temperature. Additionally, the low Ni austenitics AISI 201/EN-1.4372 and ACX 041 were also considered to cover low Ni values together with high Mn contents. This element substitutes Ni in these steel grades, among other reasons, to maintain the stability of the austenite during the transformation into strain-induced martensite during cold forming processes.

The chosen ferritic stainless steel grades are the standard AISI 430/EN-1.4016, which is characterised by 17% Cr, and the stabilised versions AISI 409/EN-1.4512, AISI 43940/EN-1.4509, ACX 700/EN-1.4003, and AISI 444/EN-1.4521, which are alloyed with Ti, Nb, or Mo and reduced C and N content to achieve better weldability, corrosion, oxidation resistance, and mechanical properties at elevated temperatures.

Finally, the choice of the duplex stainless steel is performed from the standard AISI S32205/EN-1.4462, which is fundamentally 22% Cr, 5% Ni, and 3% Mo, to the lean duplex grades AISI S32304–AISI S32304 (b)/EN-1.4362. These lasts have lower Ni and N contents without the addition of Mo; thus, their corrosion resistance is lower than the standard AISI S32205/EN-1.4462. Finally, the super duplex AISI S32507/EN-1.4410 is considered, which is characterised by 25% Cr, 7% Ni, 3% Mo, and a higher N content to improve the corrosion resistance.

### 2.2. Experimental Procedure

**Figure 1:**
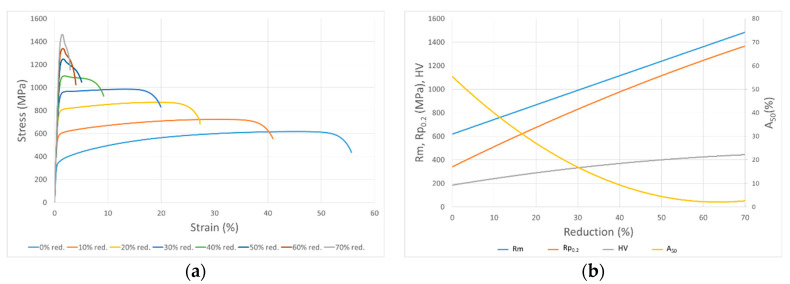
(**a**) Engineering stress–strain flow curves of A2 samples with different thickness reduction levels; (**b**) cold-rolling curves of A1 samples (*Rm* = tensile strength, *Rp*_0.2_ = yield strength at 0.2% of strain, *A*_50_ = elongation with L_0_ of 50 mm, *HV* = Vickers hardness).

Several longitudinal trips of 20 mm in width were cut from industrial hot-rolled and annealed sheets. These were cold-rolled in a Norton duo mill at lab scale with 200 mm diameter rolls and 21 rpm angular velocity, achieving seven different thickness reductions: 10, 20, 30, 40, 50, 60, and 70%. After that, the cold rolling trips were machined to obtain tensile samples, according to the standard ASTM E8/E8M. These, together with a longitudinal tensile sample extracted from the industrial hot-rolled and annealed sheet, were tensile tested until fracture according to the standard ASTM E8/E8M. From these tests, the engineering stress–strain flow curves for each steel grade, as shown in the example of Figure 1a, were obtained, and then the maximum tensile strength, *Rm*, yield strength at 0.2% of strain, *Rp*_0.2_, and elongation with an initial length of 50 mm, *A*_50_, were determined from them.

Additionally, hardness tests were carried out on all these samples according to the standards ISO 6507 and ISO 6508 for Vickers and Rockwell, respectively. Subsequently, these results of each mechanical parameter of the cold-rolled samples were represented against the cold thickness reduction for each steel grade, giving the so-called cold-rolling curves, as shown in Figure 1b. for the case of the austenitic grade A1.

Finally, these curves were used as input for the analysis with machine learning methods to obtain the models that relate the chemical composition and the thickness reduction with the mechanical properties of each stainless steel family.

### 2.3. Databases

The results obtained from the tensile and hardness tests were recorded in three different databases separately for the austenitic, ferritic, and duplex families. The number of studied samples and the obtained experimental data were different depending on the availability of steel grades from the different stainless steel families considered. In the case of the austenitic grades, a total of 130 samples were included in the database; this is the group with the highest variability of steel grades as well as production rate. Both factors make the collection of samples easier. For the ferritic types, a total of 64 samples were treated; this family has a medium diversity of steel grades, and its production rate is medium too, so the number of selected samples is lower than the austenitic case. Finally, regarding the duplex family, only 32 samples were able to be studied because this family has a reduced number of steel grades and its production is the lowest among the stainless steels under study. The number of independent variables considered in the databases was twelve; these were the cold thickness reduction and the different alloying elements. Part of the data, which represented 70% of the experimental data, was used to design and adjust the models, defined as training data, and another part, which corresponded to 15% of the available experimental data, was used for the internal validation of the same models, named validation data. Finally, the rest of the data, which represented 15% of the available data, was used to test the models (unseen data).

### 2.4. Tested Methods

Multiple comparisons of the tested models were performed to detect the best model, i.e., the model with the best generalisation capabilities for unseen data not used in the training (design) stage. The tested methods were multiple linear regression (MLR) and ANNs. The variables, which were included in each of the models, are summarised in Table 2.

In order to obtain more accurate results, a new method to select relevant features in dimensional models was proposed by Lundberg et al. [43]. This method was coined SHAP (SHapley Additive exPlanations). The SHAP method has been tested by Lee et al. [44] in order to assess the feature relevance of different variables in an industrial problem. The experimental findings demonstrated that the suggested feature importance metric based on SHAP values is more pertinent concerning forecasting performance. Moreover, an approach to diminish dataset dimensionality with minimal accuracy loss, minimising the reliance on intricate data mining, was proposed by Kumar et al. [45]. By evaluating features using their SHAP values, significant features are prioritised and those of negligible relevance to model accuracy are eliminated. According to Jeon et al. [46], in the field of steel materials, the SHAP method is used to select those characteristics to enhance the control of hardness. Other authors have also used the SHAP method for similar purposes in different applications, including Kumar et al. [47]. This method was applied to the present work to select relevant features of the different independent variables.

#### 2.4.1. Multiple Linear Regression

First, the MLR model was obtained for the prediction of the mechanical properties after a given cold strain hardening. To achieve this, as independent variables of the model, several alloying elements of the chemical composition (Ni, Cr, Cu, Mn, Mo, Si, C, N, S, Ti, and Nb) and the percentage of the thickness reduction were defined, and the mechanical parameters *Rm*, *Rp*_0.2_, *A*_50_, and *HV* were considered as dependent variables. Equation (1) expresses the MLR:

*y* = *W* · *x* + *b*, (1)

where b is the ordinate-intercept of the hyperplane *W·x*, *W* is the estimated coefficients of the MLR model, *x* is the independent variable, and *y* is the dependent variable. Matrix *W* values were estimated using the well-known least squares method with the training set of data.

#### 2.4.2. Artificial Neural Networks

**Figure 2:**
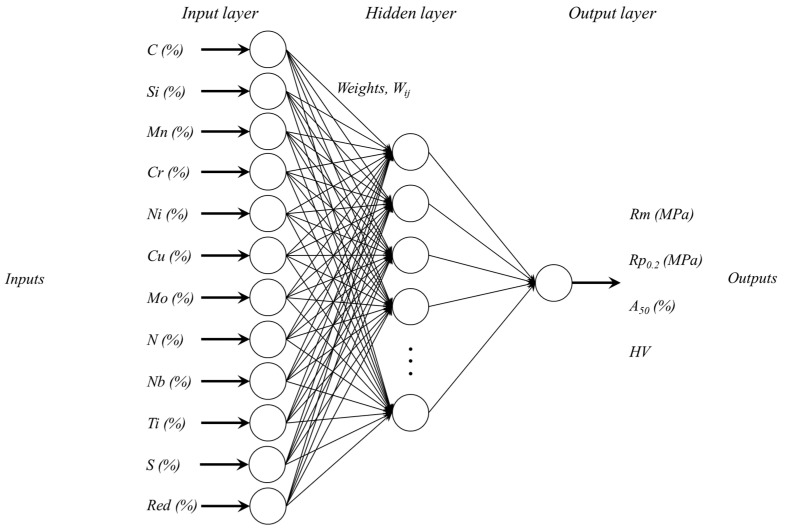
Neural network architecture modelled in this study for the prediction of the mechanical properties of cold-rolled strain-hardened stainless steel.

The main purpose of this research using ANNs is to improve and achieve better results than MLR, mainly because the elongation variable, *A*_50_, did not fit a linear behaviour. This is a model for non-linear statistical analysis in which artificial neurons are connected into an architecture that is compounded by a network of input variables, named the ‘input layer’, connected to one or more output nodes, entitled the ‘output layer’, through an intermediate layer of neurons called the ‘hidden layer’. The connection between the different neurons of the several layers is associated with various values of weights, which are adjusted to improve the predicted results of the model. In the hidden layer, sigmoid activation functions (tagsig = hyperbolic tangents) have been used, and in the output layer, a linear activation function. This kind of configuration can handle nonlinear relationships between the input and output variables [31]. The neural network architecture modelled in this study for the prediction of the mechanical properties of strain-hardened stainless steel by cold rolling is shown in Figure 2.

Equation (2) shows a typical ANN, which is composed of non-linear activation functions *f*_1_ and *f*_2_, where *W*_1_ or *W_ih_* are the weights from the input layer to the hidden layer, *W*_2_ or *W_ho_* are the weights from the hidden layer to the output layer, and *f_n_* are continuous and derivable functions with bias in the neurons or every hidden layer. Moreover, *b*_1_ and *b*_2_ are bias vectors. Bias nodes help networks solve more types of problems by allowing them to employ more complex logic gates.

*y*_*predicted*_ = *f*_2_ × (*W*_2_ × (*f*_1_(*W*_1_ × *x* + *b*_1_) + *b*_2_)), (2)

The modelled network for this study was trained using the standard backpropagation algorithm [25] with the Levenberg–Marquardt optimisation method.

The inputs defined for this ANN model were the alloying elements of the chemical composition (Ni, Cr, Cu, Mn, Mo, Si, C, N, S, Ti, and Nb) and the percentage of the applied cold thickness reduction, and, as output neurons, the mechanical parameters *Rm*, *Rp*_0.2_, *A*_50_, and *HV*. Neurons were connected to all previous layers by weight connections. During the training process, each predicted output was compared with the real value, and the calculated error was backpropagated to the hidden and input layers to adjust the weights and minimise the *MSE* error. Different numbers of neurons in the hidden layer (1, 5, 20, and 50) were used during the training stage to study the optimal structure of the network together with the application of the early stopping procedure using the internal validation set of data. The ANN architectures characterised by fully connected multi-layers almost always have too large a parameter space, being prone to overfitting. A widely used procedure to fight this drawback of the ANNs is early stopping due to its simplicity to be understood and implemented [48,49]. In this work, the internal validation was performed using 15% of the data, and a further 15% was applied to the test set.

#### 2.4.3. Shapley Relevance Determination Method

SHAP (Shapley additive explanations) is a technique used in the field of machine learning to elucidate the outcomes of any machine learning model. It assigns a significance score to each feature in a specific prediction. The importance of each feature is derived by evaluating its contribution across all conceivable combinations of features, providing a comprehensive understanding of their impact on predictions. This approach facilitates the interpretation of complex models, offering insights into the contribution of each feature to the ultimate prediction [43,45,47].

The value function *v* defines the Shapley value of the *i*th feature for the query point *x*, as shown through Equation (3), where *M* is the number of all features, *m* is the set of all features, |*S*| is the cardinality of the set *S*, or the number of elements in the set *S*, and *v_x_(S)* is the value function of the features in a set *S* for the query point *x*. The value of the function indicates the expected contribution of the features in *S* to the prediction for the query point *x*.

(3) $$\varphi_{i}\left(v_{x}\right)=\frac{1}{M}\sum_{S\subseteq m\backslash \left\{i\right\}}\frac{v_{x}\left(S\cup \left\{i\right\}\right)-v_{x}(S)}{\frac{\left(M-1\right)!}{\left|S\right|!\left(M-\left\lceil S\right\rceil -1\right)!}}$$

## 3. Results

Following the experimental procedure described above, two quality indexes (*R* and *MSE*) have been computed for test sets in a resampling procedure. This procedure consists of 20 random replications of training–validation–test phases comparing the average values of the test set, which are collected in Table 3 for every dependent variable. Therefore, these values were computed for test sets to assess the generalisation capabilities of the models. The best model for each mechanical parameter and stainless steel grade is chosen according to the values of *MSE* and *R*; these appear in bold in Table 3. 

An ANOVA test was applied in order to compare the differences among group means in a sample. ANOVA compares the variance within groups to the variance between groups. On the other hand, the Friedman test is a non-parametric statistical test used to detect differences in treatments across multiple test attempts when data does not follow a normal distribution. ANOVA/Friedman tests assess whether there are differences among groups but do not identify which specific groups are different. Thus, post hoc tests or pairwise comparisons can be conducted for this purpose. Specifically, the Bonferroni correction [29] is used to adjust significance levels when conducting multiple statistical tests simultaneously and allow us to determine which the best model is.

**Figure 3:**
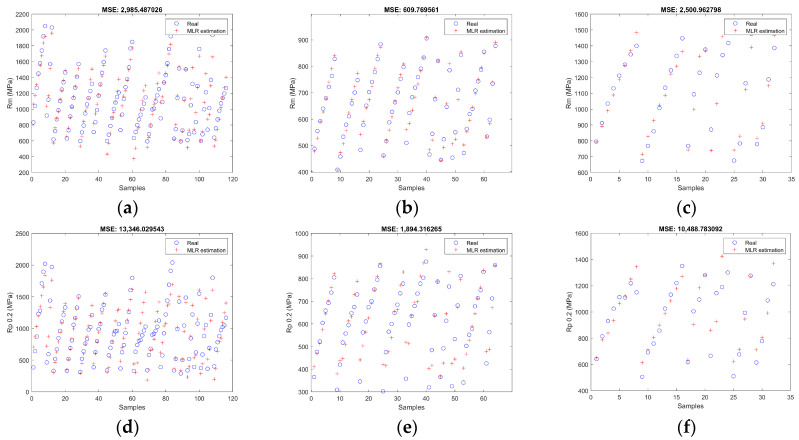

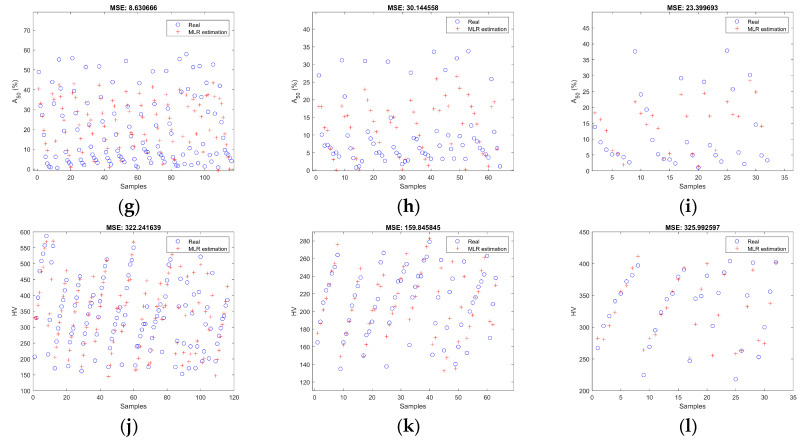
Mechanical property estimation plot for the best ANN model. *Rm* (MPa): (**a**) austenitics; (**b**) ferritics; (**c**) duplex. *Rp*_0.2_ (MPa): (**d**) austenitics; (**e**) ferritics; (**f**) duplex. *A*_50_ (%): (**g**) austenitics; (**h**) ferritics; (**i**) duplex. *HV*: (**j**) austenitics; (**k**) ferritics; (**l**) duplex.

Figure 3 collects some numerical results of *MSE* for each type of stainless steel and each one of its properties. This depicts the mean-square error for the best model on a certain output using the best configuration of hidden units. The “*x*” axis represents the number of each analysed sample, and the “y” axis represents the experimental value (blue circle) and the estimated one by ANNs (red cross) for each one of them. The higher the deviation between the blue circles and red crosses, the higher the *MSE* of that model for the steel grade and mechanical property given.

**Figure 4:**
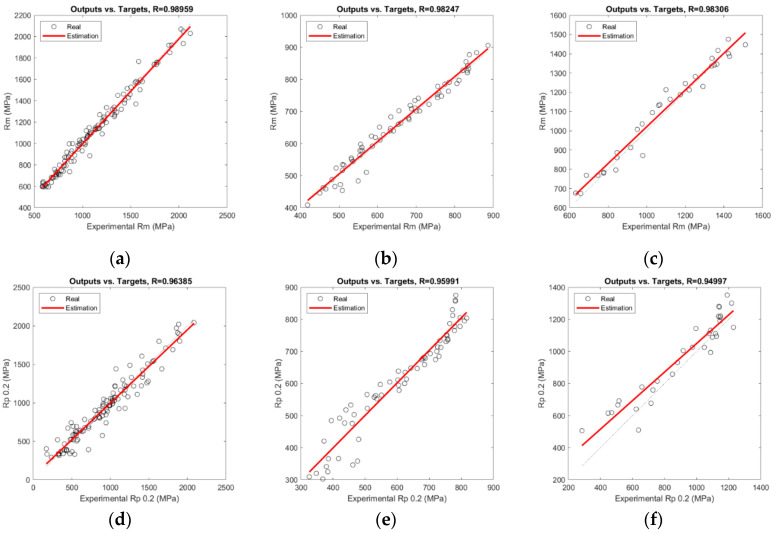

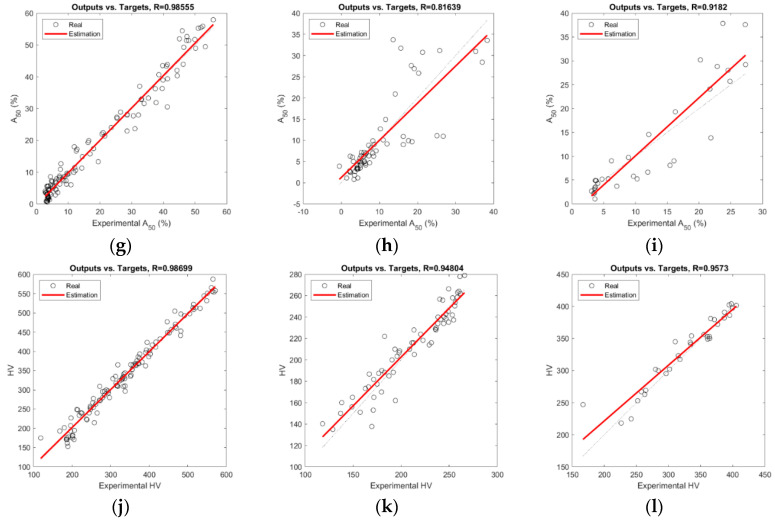
Correlation plots comparing experimental and predicted mechanical properties. *Rm* (MPa): (**a**) austenitics; (**b**) ferritics; (**c**) duplex. *Rp*_0.2_ (MPa): (**d**) austenitics; (**e**) ferritics; (**f**) duplex. *A*_50_ (%): (**g**) austenitics; (**h**) ferritics; (**i**) duplex. *HV*: (**j**) austenitics; (**k**) ferritics; (**l**) duplex.

Figure 4 shows the correlation between the experimental and predicted values of each output for the training set and steel family. A red line points out the real correlation between the experimental and estimated values. A dashed black line shows the theoretical best fit between both sets of data, which means that the estimated values are just like the experimental ones. The higher the *R* value of the model for a steel grade and mechanical property, the better the fitting between both lines. In the graphs in this figure, all the samples belonging to the test set have been considered and computed. The procedure has been repeated until all the samples have been selected in the test set at least once. The final result of *R* and *MSE* is computed using the whole set of samples as the test set.

**Figure 5:**
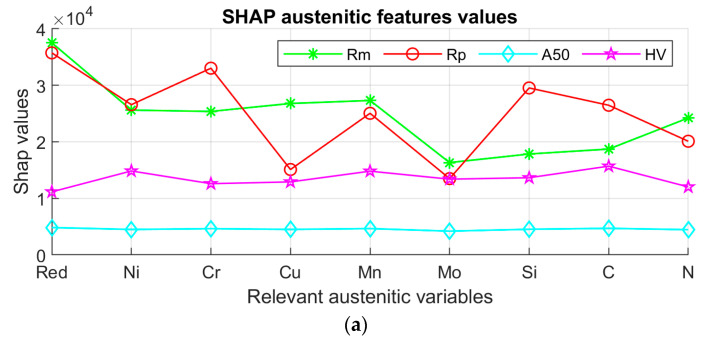

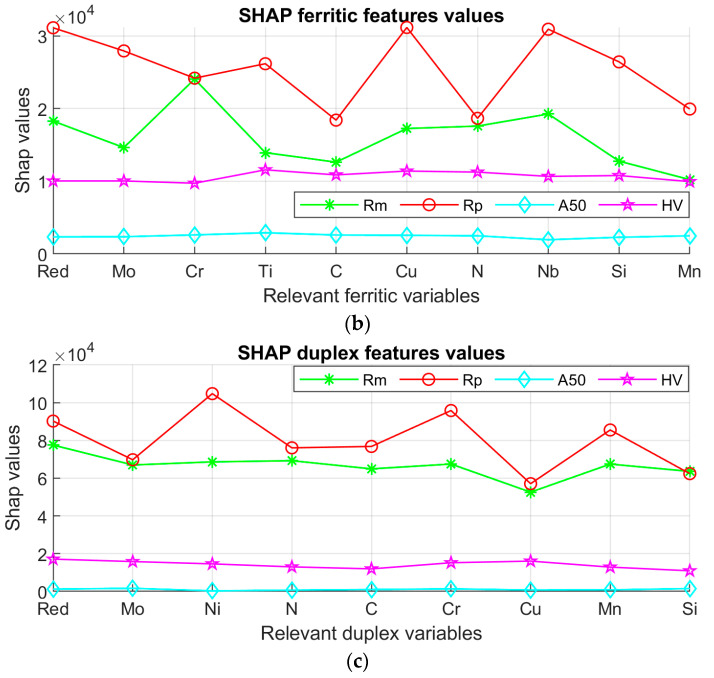
Experimental results of the SHAP method for the austenitic (**a**), ferritic (**b**), and duplex grades (**c**).

Figure 5 shows the results of the SHAP method. Greater SHAP values mean a higher impact or influence of the corresponding composition variable on the output. Specifically, in Figure 5, it is observed that there is greater variability in the relevance of the chemical composition in the *Rm* and *Rp* properties, especially in the austenitic and ferritic steels. In duplex steels, there is variability in *Rp* and a more constant relevance between the different chemical elements in the case of *Rm*. Likewise, in the *A*_50_ and *HV* properties, there are no elements with significantly greater or lesser relevance than others. And in the case of *A*_50_ and *HV* in general, the significance values calculated by the SHAP method are lower than in the case of *Rm* and *Rp* in all steels.

## 4. Discussion

The criteria that are followed to define the quality of the designed models are that the lower the *MSE* and the higher the *R*, the better the prediction.

First, with respect to the MRL model, it can be extracted from Table 3 that this showed reasonably good *R* values (over 0.93) for *Rm*, *Rp*_0.2_, and *HV* for the three stainless steel families. In consequence, the relationship between the chemical composition and thickness reduction with those three mechanical properties is almost linear, and then the MRL model can be an easy and fast solution to the studied modelling. Despite that, this trend is not the same for ductility, which exhibited *R* values ranging from 0.74 to 0.91. This last point makes it necessary to search for another model that is able to predict ductility with better results than those achieved by the MRL model.

In this sense, the comparison of the two quality indexes, *R* and *MSE*, in the studied models (Table 3) shows that ANNs outperformed MLR in all cases, with an *R* higher than 0.95 for most parameters. Moreover, the error is significantly reduced in ANN models, which means that ANNs make better predictions than MLR. In this work, the resulting values for the ductility parameter *A*_50_ were significantly improved for most of the ANN models. Normally, an enhanced *R* goes together with an enhanced *MSE*. However, there are exceptions where outputs with a higher *R* also have a higher *MSE*. This can be observed in Table 3 through the ANN model of one hidden unit for the *Rp*_0.2_ and *HV* parameters of the duplex stainless steel family: these obtain the best *R* with a value of 0.95 and 0.96, respectively, but worse *MSE* than the MRL models of a single hidden unit. 

The results showed that an ANN model with fewer neurons (between one and five) is the best choice for obtaining better generalisation capacities and better fitting to the test data. In this modelling process, there appears to be a high degree of linearity between inputs and desired outputs. In the case of austenitic and ferritic stainless steel, the best result is normally obtained by an ANN model with five hidden neurons. Thereafter, it is not necessary to use a more complex model with more neurons. The results reached lower values in the correlation coefficient for the MLR models and close to unity in most cases for the ANN models. These results show the suitability of the proposed approach.

The analysis of the average values of *MSE* and *R* collected in Table 3 for the ANN model of each mechanical property and stainless steel family makes it evident that ANNs perform significantly better in modelling the mechanical parameters of the austenitic stainless steel family compared to the rest of the steels and to MLR. The correlation coefficient of the ANN models for the *Rm*, *A*_50_, and *HV* of the austenitics is 0.98, while this index for the *Rp*_0.2_ of the same family is 0.96. The MSEs of all the modelled parameters are considerably reduced with the application of ANNs, up to 5 to 10 times with respect to the obtained values with MLR. These results confirm the hypothesis that ANNs improve the modelling capacity of these models.

The same tendency is observed for the ferritic grades, in which parameters *Rm* and *Rp*_0.2_ are quite well-modelled with ANNs, as their correlation coefficients are above 0.96. However, the *A*_50_ and *HV* mechanical properties are not as well modelled and have correlation coefficients of 0.82 and 0.95, respectively. 

Regarding the duplex family, according to the obtained *R* values, which are higher than 0.95 for most parameters, an equivalent behaviour is obtained when the mechanical properties are modelled by ANNs. Nevertheless, the MSE for the parameters *Rp*_0.2_ and *HV* is higher in the case of the ANN models compared to the MRL model. This exception is probably a consequence of two factors: the smaller amount of data available for this family with respect to the austenitic and ferritic grades, and the two-phase microstructure of the duplex steels, which is formed by austenite and ferrite in an approximately 50:50 proportion. In relation to this last point, the level of alloying elements influences the stability of the austenitic phase to be transformed into strain-induced martensite when the duplex steel is cold-formed. This makes the duplex microstructure more similar to the austenitic or ferritic one, making it difficult to predict the cold strain hardening of this family compared to the others considered in this work.

Secondly, concerning the effect of the independent variables considered in this work of the modelling of the strain-hardened mechanical properties, the cold thickness reduction (*Red*) is the most relevant feature for the three stainless steel families. This is explained through the best fitting of the *Rm*, *Rp*_0.2_, and *HV* models for the higher values of these mechanical parameters when the cold thickness reduction is increased, as it is analysed in graphs “a–f” and “j–k” in Figure 3 and Figure 4. 

However, a higher cold thickness reduction results in lower ductility and better *A_50_* model fitting. This is justified by the nearly asymptotic behaviour of this dependent variable, which tends to zero when the cold thickness reduction is above 40%, making the modelling better when the *A*_50_ is low. Additionally, a significant loss of ductility is produced on stainless steels when small cold thickness reductions are applied. Thereupon, this drop of the elongation when the stainless is cold-formed together with the asymptotic trend makes the modelling of this parameter more difficult, as shown by the lower *R* values in graphs “h” and “i” of Figure 4 regarding the rest of the modelled mechanical parameters, only reaching a *R* equal to 0.8164 in the ferritic family and 0.9182 for the duplex grades. A database with more samples (a wider database) could probably provide more reliable results in those cases where the prediction does not reach adequate values.

In addition to the influence of the *Red* independent variable on the developed models, several alloying elements (such as Ni, Cr, N, and C) also have an important role in them. The austenitic stainless steels are more sensitive to these variables than the ferritic grades. This is because the austenitics are transformed into strain-induced martensite when they are subjected to cold forming. Therefore, the *MSE* of the austenitic family is higher than that of the ferritic one, which is less dependent on chemical composition because no phase transformation occurs during cold-forming processes. Another factor that contributes to the higher *MSE* for the austenitic steels is the wider range of chemical compositions analysed compared to the ferritic ones. One simple way to show this is by comparing the maximum and minimum values of *Cr_eq_* and *Ni_eq_* (Equations (3) and (4) from Schaeffler [50]) for both families; while the range of *Cr_eq_* is quite similar between the austenitics and ferritics (*Cr_eq max_*: *Cr_eq min_*, 25.85: 16.61 against 20.62: 11.76 for austenitics and ferritics, respectively), the variation of the *Ni_eq_* is greater for the austenitics than the ferritics (Ni*_eq max_*: *Ni_eq min_*, 25.85: 8.76 against 2.42: 0.809 for austenitics and ferritics, respectively). As a result, taking into account the *MSE* values, the ferritic family is modelled better than the other families considered in this study. This can be observed in graphs “a”, “d”, and “j” of Figure 3 of the austenitic grades, where *MSE* values are higher than those in graphs “b”, “e”, and “k” in Figure 3, corresponding to the ferritic families.

*Cr_eq_ = Cr + Mo + 1.5·Si + 0.5·Nb*, (4)

*Ni_eq_ = Ni + 30·C + 0.5·Mn*, (5)

Figure 5 shows the results of the SHAP relevance analysis. In the case of the austenitic steels, the highest significance for both *Rm* and *Rp* is found in the reduction variable, as well as in Nickel (Ni), Chromium (Cr), Silicon (Si) and Manganese (Mn). In the case of ferritics, the highest relevance is found in the reduction variable, Molybdenum (Mo), Copper (Cu), Niobium (Nb), and Chromium. In the case of the duplex type, the highest relevance is found in Nickel, Chromium, reduction and Manganese, being more similar to the austenitic steels, although the changes in the composition of the main elements affect more strongly since larger SHAP relevance values are appreciated.

Finally, based on the analysis of the results, it can be inferred that modelling the mechanical properties of duplex steels is a challenging task. This is because different metallurgical phenomena can occur depending on the level of alloying grade in this family, causing the material to behave more like either austenitic or ferritic steel, or neither of these families, and making its modelling difficult. Additionally, there is a limited amount of data available for duplex steels, as has been described previously, which further complicates the modelling process.

## 5. Conclusions

An artificial neural network approach, which is compared to MLR, is used to predict the mechanical properties of cold strain-hardened stainless steels as a function of the chemical composition and cold thickness reduction. The main conclusions achieved are the following:

- The relationship between the chemical composition and thickness reduction with most of the mechanical properties analysed (*Rm*, *Rp*_0.2_, and *HV*) is almost linear. Thus, the MLR model can be adequate as an economical and fast method to model this process.
- Nevertheless, the ductility parameter does not follow a linear relationship with the chemical composition and the thickness reduction.
- To overcome the limitation of the MRL model for the prediction of the ductility parameter and to improve the accuracy for the rest of the mechanical properties, it has been found that ANNs are more effective than MLR. ANNs produce lower mean squared error and higher correlation coefficient values.
- Therefore, the cold-rolling curves can be modelled by ANNs without the need for complex models that require more than five neurons.
- The main independent variable in the designed models is the percentage of cold thickness reduction, which is consistent across the three stainless steel families.
- The austenitics are more susceptible to the influence of the alloying elements. This is due to their effect on the stability of the austenite phase and also to the higher range of chemical composition analysed in this work.
- In general, it is observed that higher values of *Rm*, *Rp*_0.2_, and *HV* lead to better model fitting, while lower values of *A*_50_ result in better model prediction.
- By applying the SHAP method, it can be concluded that the variables *Rm* and *Rp* are more sensitive to changes in chemical composition as well as to the variable reduction in all steels. And the variables *A*_50_ and *HV* seem to be less sensitive to changes in chemical composition.

This study demonstrates that machine learning techniques can establish correlations between the chemical composition, cold thickness reduction, and mechanical properties of materials. These correlations can be applied in the industry to reduce manufacturing costs, not only during the design phase of new alloys but also during the selection of materials and definition of cold-forming process conditions. All in all, this approach provides a cost-effective solution for optimising material properties in the manufacturing process.

## Figures and Tables

**Table 1:** Identification and chemical composition (in weight %) of the selected industrial hot-rolled and annealed sheets. The grades that do not have a standard designation are named by the internal Acerinox ID (in brackets) [41].

ID.	AISI [42]	EN [21]	C	Si	Mn	Cr	Ni	Cu	Mo	N	Nb	Ti	S
A1	301	1.4310	0.105	0.86	1.24	16.78	6.71	0.20	0.34	0.069	0.013	0.008	0.0010
A2	304	1.4301	0.029	0.38	1.75	17.89	8.06	0.42	0.25	0.074	0.011	0.010	0.0010
A3	316L	1.4404	0.022	0.35	1.38	16.70	10.28	0.32	2.26	0.053	0.021	0.009	0.0020
A4	(ACX 041)	-	0.083	0.43	9.62	15.95	1.46	0.19	0.03	0.160	0.005	0.019	0.0020
A5	310S	1.4845	0.045	0.55	1.36	24.60	19.15	0.18	0.01	0.025	0.011	0.003	0.0010
A6	201	1.4372	0.076	0.50	7.06	16.12	4.32	0.58	0.18	0.083	0.008	0.004	0.0010
A7	904L	1.4539	0.019	0.42	1.50	19.58	24.52	1.40	4.25	0.015	0.022	0.006	0.0010
A8	309S	1.4833	0.044	0.49	1.63	22.37	13.94	0.28	0.49	0.058	0.007	0.013	0.0010
F1	430	1.4016	0.055	0.36	0.49	16.12	0.38	0.10	0.02	0.034	0.006	0.007	0.0040
F2	409	1.4512	0.011	0.52	0.45	10.97	0.26	0.08	0.02	0.009	0.005	0.196	0.0010
F3	S43940	1.4509	0.017	0.53	0.39	17.56	0.35	0.06	0.02	0.014	0.399	0.139	0.0010
F4	(ACX 700)	1.4003	0.015	0.73	0.88	10.88	0.63	0.08	0.02	0.010	0.007	0.142	0.0020
F5	444	1.4521	0.016	0.45	0.30	17.85	0.20	0.09	1.88	0.019	0.440	0.156	0.0010
D1	S32205	1.4462	0.021	0.41	1.40	22.65	5.39	0.12	3.18	0.167	0.011	0.027	0.0010
D2	S32304	1.4362	0.020	0.52	1.26	22.43	4.16	0.25	0.17	0.100	0.006	0.028	0.0007
D3	S32304 (b)	1.4362	0.016	0.46	1.33	23.90	4.30	0.29	0.55	0.177	0.006	0.007	0.0004
D4	S32507	1.4410	0.014	0.33	0.76	25.00	6.80	0.25	3.75	0.265	0.029	0.010	0.0010

**Table 2:** Identification of variables used in both models.

Variables	Symbol	Description
Input	Ni, Cr, Cu, Mn, Mo, Si, C, N, S, Ti, and Nb	Concentration of each alloying element (%)
	Red	Cold thickness reduction (%)
Output	Rm	Tensile strength (MPa)
	Rp_0.2_	Yield strength at 0.2% (MPa)
	A_50_	Elongation (%)
	HV	Vickers hardness

**Table 3:** Comparison between methods: MLR and ANNs for 20 repetitions in the three tests. Numbers in bold are the best results of *MSE* and *R* for each analysed model.

Test	Stainless Steel Family	Outputs	Quality Index	MLR	ANNs			
					Number of Hidden Units (Input–Hidden–Output)			
					1	5	20	50
Test 1	Austenitic	*Rm*	*MSE*	7872.01	4761.76	**2985.49**	6693.91	28,244.11
			*R*	0.9716	0.9832	**0.9896**	0.9769	0.9280
		*Rp*_0.2_	*MSE*	21,878.03	30,108.63	**13,346.03**	17,316.02	46,407.56
			*R*	0.9379	0.9168	**0.9639**	0.9557	0.9055
		*A*_50_	*MSE*	51.64	**8.63**	10.96	36.33	69.74
			*R*	0.9097	**0.9856**	0.9819	0.9435	0.8967
		*HV*	*MSE*	1280.09	1538.13	**322.24**	1456.61	3331.30
			*R*	0.9471	0.9377	**0.9870**	0.9483	0.9040
Test 2	Ferritic	*Rm*	*MSE*	1098.17	731.15	**609.76**	2076.57	3902.08
			*R*	0.9659	0.9784	**0.9825**	0.9406	0.9032
		*Rp*_0.2_	*MSE*	2602.26	**1894.31**	4334.46	4493.21	6835.87
			*R*	0.9460	**0.9599**	0.9345	0.9263	0.9032
		*A*_50_	*MSE*	40.43	36.61	**30.14**	92.61	137.36
			*R*	0.7357	0.8025	**0.8164**	0.7806	0.6938
		*HV*	*MSE*	189.82	174.92	**159.84**	350.68	697.01
			*R*	0.9315	0.9442	**0.9480**	0.9007	0.8441
Test 3	Duplex	*Rm*	*MSE*	3540.29	4157.56	**2500.96**	10,527.59	16,801.74
			*R*	0.9726	0.9712	**0.9831**	0.9503	0.9019
		*Rp*_0.2_	*MSE*	6543.80	**10,488.78**	10,472.97	8143.71	21,569.70
			*R*	0.9473	**0.9499**	0.9464	0.9371	0.9051
		*A*_50_	*MSE*	26.97	**23.39**	20.49	31.86	32.95
			*R*	0.8882	**0.9182**	0.9166	0.8708	0.8784
		*HV*	*MSE*	295.96	**325.99**	352.27	462.63	1739.17
			*R*	0.9490	**0.9573**	0.9455	0.9195	0.8206

## Data Availability

The raw/processed data required to reproduce these findings cannot be shared at this time due to legal or ethical reasons.
